# Validation of DESS as a DNA Preservation Method for the Detection of *Strongyloides* spp. in Canine Feces

**DOI:** 10.3390/ijerph14060624

**Published:** 2017-06-09

**Authors:** Meruyert Beknazarova, Shelby Millsteed, Gemma Robertson, Harriet Whiley, Kirstin Ross

**Affiliations:** 1 School of the Environment, Flinders University, GPO Box 2100, Adelaide 5001, Australia; shelby.millsteed@gmail.com (S.M.); harriet.whiley@flinders.edu.au (H.W.); Kirstin.Ross@flinders.edu.au (K.R.); 2 Melbourne Pathology, Collingwood and James Cook University, Collingwood, VIC 3066, Australia; gemmajrobertson@gmail.com

**Keywords:** *Strongyloides stercoralis*, *Strongyloides ratti*, *Strongyloides*, real-time PCR, DESS, DNA preservation, DNA degradation, canine feces

## Abstract

*Strongyloides stercoralis* is a gastrointestinal parasitic nematode with a life cycle that includes free-living and parasitic forms. For both clinical (diagnostic) and environmental evaluation, it is important that we can detect *Strongyloides* spp. in both human and non-human fecal samples. Real-time PCR is the most feasible method for detecting the parasite in both clinical and environmental samples that have been preserved. However, one of the biggest challenges with PCR detection is DNA degradation during the postage time from rural and remote areas to the laboratory. This study included a laboratory assessment and field validation of DESS (dimethyl sulfoxide, disodium EDTA, and saturated NaCl) preservation of *Strongyloides* spp. DNA in fecal samples. The laboratory study investigated the capacity of 1:1 and 1:3 sample to DESS ratios to preserve *Strongyloides ratti* in spike canine feces. It was found that both ratios of DESS significantly prevented DNA degradation compared to the untreated sample. This method was then validated by applying it to the field-collected canine feces and detecting *Strongyloides* DNA using PCR. A total of 37 canine feces samples were collected and preserved in the 1:3 ratio (sample: DESS) and of these, 17 were positive for *Strongyloides* spp. The study shows that both 1:1 and 1:3 sample to DESS ratios were able to preserve the *Strongyloides* spp. DNA in canine feces samples stored at room temperature for up to 56 days. This DESS preservation method presents the most applicable and feasible method for the *Strongyloides* DNA preservation in field-collected feces.

## 1. Introduction

*Strongyloides stercoralis* is a gastrointestinal human parasitic nematode whose lifecycle includes both free-living and parasitic forms [1]. The environmental phase comprises of one free-living generation with long-lived infective larvae (L3) [1]. Globally, it is estimated that there are 370 million people infected with *Strongyloides* spp. [2]. However, there is limited knowledge regarding the parasites’ survival in the environment and limited public health strategies for controlling the disease [3]. For both clinical (diagnostic) and environmental evaluation, it is essential to have a reliable method for *Strongyloides* spp. detection in both human and non-human fecal samples. 

The real-time polymerase chain reaction (PCR) is a nucleic acid detection-based technique which has been shown to have high sensitivity and specificity for pathogen detection (e.g., *Strongyloides* spp.) in fecal samples [4,5]. The ability to use preserved or frozen samples for PCR makes it a convenient method for field-based samples [4,6], and preferable to culture detection which relies on live organisms [7]. The success of PCR as a detection method is generally dependent on whether nuclear or mitochondrial DNA is amplified, the number of base pairs targeted by PCR, the presence of PCR inhibitors, and the preservation of the targeted DNA in the samples [8]. As such, it is imperative to preserve the DNA and increase the feasibility of using fecal DNA. One of the biggest challenges to this method is DNA degradation during postage time from rural and remote areas to the laboratory. This degradation is due to components of feces that rapidly degrade DNA [9], and is compounded by the fact that it is unfeasible to refrigerate samples during the travel time from rural locations and it is often impossible to post liquids with alcohol-based preservatives.

DNA in biological samples degrades as cells lyse due to decomposition processes, and free DNA is hydrolyzed by DNAses produced by microorganisms in the sample [10,11]. Studies suggest that positive PCR results in fecal samples significantly decline after three days from collection, which is well below the expected collection and transport time for field-collected samples [12]. While a number of reasonably successful DNA preservation methods currently exist, their applicability to field-based collection and transportation from rural areas is lacking. An ideal DNA preservative will be easy, safe, and inexpensive to utilize, transport, and store [13]. Cryopreservation is considered to be the most effective method for long-term DNA preservation, but it is difficult to acquire the equipment to achieve this in rural areas, and strict regulations prevent cryopreserved samples being transported by air [13,14]. Ethanol is cheap and readily available, but does not preserve optimally at room temperature [12,15], and may require additional processing such as sample homogenization, subsampling, and changing the ethanol after a few days of storage, which may be unfeasible when dealing with large numbers of samples [13]. Furthermore, ethanol is a hazardous substance and restrictions apply when transporting by air [15]. Other methods are either ineffective, or prohibitively expensive—as is the case with proprietary solutions [14]. A field-applicable method, which is effective, easy, safe, and inexpensive should be developed for studies involving environmental samples collected from rural areas.

DESS (dimethyl sulfoxide, disodium EDTA, and saturated NaCl) has been previously shown to be highly effective in long-term DNA preservation of different tissues at room temperature [8,13,14,15,16]. However, this is the first study that has developed and validated a method of DNA preservation using DESS for the subsequent detection of *Strongyloides* spp. in fecal samples. The method was first tested in the laboratory using fresh non-infected dog feces spiked with *Strongyloides ratti,* as a model organism used to determine the effectiveness of 1:1 and 1:3 sample: DESS ratios for DNA preservation at room temperature. This was then validated by using the method on field-collected dog fecal samples for *Strongyloides* spp. detection. 

## 2. Materials and Methods 

### 2.1. DESS Protocol 

100 mL of deionized water was added to 46.53 g EDTA disodium salt and stirred. Then, 1 M NaOH was added while heating at 30 °C until the pH reached 7.5 and the EDTA disodium salt had dissolved. A 400-mL aliquot of the solution was created and 100 mL of DMSO was added for a final DMSO concentration of 20% and EDTA disodium salt concentration of 0.25 M. Finally, 50 g NaCl was added to the solution.

### 2.2. Assessing the Ability of DESS to Preserve Strongyloides ratti DNA in Feces 

Fresh dog fecal samples were collected from a non-infected dog and utilized within 24 h of collection. Fresh feces from rats infected with *S. ratti* via subcutaneous penetration were collected 7–10 days post infection and donated by the Center for Infectious Diseases and Microbiology, Westmead Hospital, NSW, Australia; this was used to spike the canine feces at a 1:9 rat to canine feces ratio. 

The spiked feces (canine and rat combined) was then aliquoted and either treated with 1:1 or 1:3 sample to DESS ratio or no DESS (for the no-treatment control). All samples were conducted in duplicate for each treatment/control and DNA extraction time point (day 0, 3, 7, 14, 28 and 56). A negative control was also included containing non-infected fresh canine feces dissolved in DESS solution at a 1:1 ratio. The negative controls were conducted in duplicate for each DNA extraction time point (day 3, 14 and 56). All samples were stored in sealed containers at room temperature prior to DNA extraction using the method described below. 

### 2.3. PCR Positive and Negative Controls 

PCR positive controls were created using fresh dog fecal samples spiked with feces from rats infected with *S. ratti*, and PCR negative controls were created using non-infected canine feces. The DNA from these positive and negative controls were extracted immediately.

### 2.4. DNA Extraction

Prior to DNA extraction, samples containing DESS were centrifuged for 3 min at 3000× *g* rpm at the Orbital 400 Clements (Phoenix, Lidcombe, Australia). The supernatant was removed, and approximately half a milligram of the remaining pellet was exposed to further DNA extraction using the PowerSoil DNA extraction kit as per the manufacturer’s protocol (QIAGEN, Hilden, Germany). A slight modification to the methods was introduced, comprising an incubation of the sample at 56 °C overnight, after the cell lysis step [17,18].

### 2.5. Field Collection of Canine Feces 

A total of 37 canine samples were collected from eight different locations across Australia. Two grams of dog feces were collected into the plastic tube containing 6 mL of DESS solution. The samples were kept at room temperature up to 30 days until being processed at the laboratory. 

The sample collection was approved by the Social and Behavioral Research Ethics Committee # 6852. 

### 2.6. Real-Time PCR Conditions for Strongyloides *spp.*

The real-time PCR assay was adopted from Verweij et al. (2009) and Sultana et al. (2013), using the *S. stercoralis* species-specific primers and probes (the F primer is 100% homologous to other *Strongyloides* species) targeting a 101 base pair region of 18S rRNA gene (GenBank accession No. AF279916) [4,5]. The 20 µL reaction contained 10 µL of Supermix (SSoAdvanced, Universal Probes Supermix, Foster City, CA, USA), 1 µL of primers and probe mixture (Stro18S-1530F 5′-GAATTCCAAGTAAACGTAAGTCATTAGC-3′; Stro18S-1630R 5′-TGCCTCTGGATATTGCTCAGTTC-3′ and probe Stro18S-1586T FAM-5′-ACACACCGGCCGTCGCTGC03′-BHQ1) [4], 4 µL of deionised H_2_O, and 5 µL of DNA template. The cycling conditions included an initial hold at 95 °C for 15 min, followed by 40 cycles consisting of 95 °C for 15 s and 60 °C for 30 s. All PCR reactions were performed in triplicate using the Corbett Rotor-Gene 6000 machine (QIAGEN, Hilden, Germany). Each PCR run contained a positive, negative, and non-template control (NTC). To determine the presence of environmental inhibitors, all samples were tested at pure and 1 in 10 dilution of the DNA extract into nuclease-free water. If the cycle threshold (Ct) value for the pure DNA extract was 3.3 higher the Ct value of the 1 to 10 dilution of DNA extract, then the pure DNA was assumed to be inhibited by the environmental inhibitors and the diluted DNA extracts were used [19].

A sample was considered positive when the Ct value was lower the mean negative Ct minus 2.6 standard deviations of a mean negative control Ct. Positive samples were amplified in every PCR reaction (Ct 20.50–24.65).

### 2.7. Statistical Analysis

Statistical analysis was performed using SPSS (SPSS Inc., Chicago, IL, USA). Related Samples Wilcoxon Signed Rank Test was performed to compare the means of replicates within each treatment. One-Sample Kolmogorov-Smirnov Test was used to check the data distribution. Paired-sample *t*-test was performed to compare the DESS-treated samples with control samples, and DESS 1:1 to DESS 1:3 treatments. 

The means of duplicates of each treatment sample (DESS 1:1, DESS 1:3, and control) were not statistically significantly different (*p* > 0.05). This shows the replicability of the PCR runs and rules out experimental errors associated with pipetting. One-Sample Kolmogorov-Smirnov Test was further performed for each of the treatments (duplicates) at each measured day. The data distribution was found normal within the treatments and means of the treatments were further used for the graphs. 

## 3. Results

### 3.1. Effect of DESS Solution on the Preservation of Strongyloides ratti DNA over Time

**Figure 1:**
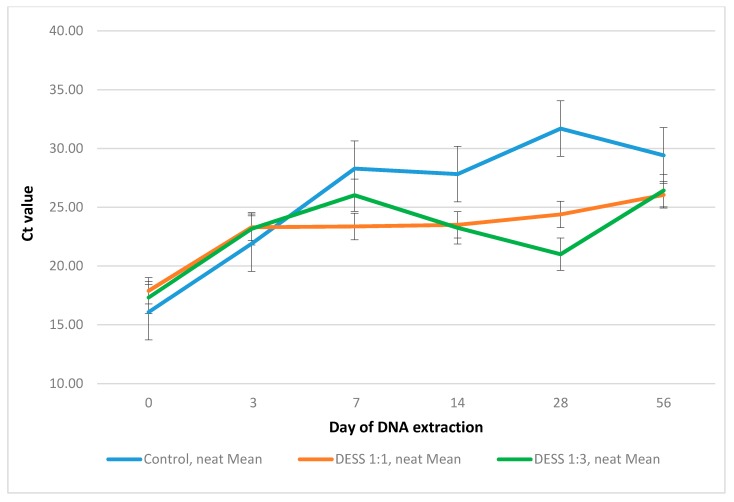
Performance of 1:1 and 1:3 ratios of *Strongyloides ratti* spiked fecal sample to DESS (dimethyl sulfoxide, disodium EDTA, and saturated NaCl) and a no-treatment control over time.

DESS-treated samples preserved DNA better than no-treatment controls for 56 days. Control and DESS-treated groups were statistically significantly different (*p* = 0.000), while there was no significant difference between DESS 1:1 and DESS 1:3 treated samples (*p* = 0.752). Figure 1 shows the mean Ct values for 1:1 and 1:3 sample to DESS ratios and a no-treatment control for each DNA extraction time point. 

Undiluted DNA extracts were consistently amplified better (corresponding to lower Ct values) compared to 1 to 10 DNA dilutions, indicating that there was no inhibition observed. DESS negative control samples appeared to be negative consistently over the incubation period, indicating that there was no cross-contamination between the samples during the incubation period. 

Significant degradation of the *S. ratti* DNA in no-treatment samples was observed after two weeks of storage at room temperature. The 1:3 treated samples showed Ct values slightly and steadily decreasing from day 7 to day 28. The heterogeneity of the feces matter with uneven distribution of different inhibitors (bilirubins, bile salts, complex carbohydrates) [9] might affect the DNA amplification by PCR. 

### 3.2. DESS Preservation Effect in Field-Collected Canine Feces 

From the 37 canine fecal samples collected and stored in a 1:3 sample:DESS ratio for up to 30 days at room temperature, there were 17 positive for *Strongyloides* DNA.

## 4. Discussion

This study demonstrated that DESS in both 1:1 and 1:3 sample to DESS ratios could preserve *S. ratti* DNA in canine feces for up to 56 days at room temperature. It was important to identify the optimum concentration of DESS, as higher concentrations might result in its active components releasing inhibiting properties and interfering with PCR amplification. However, no statistically significant difference was observed with the two different DESS concentrations. Being a nonproprietary solution, cost-effective, not resource intensive, and non-flammable, DESS presents a feasible option for DNA preservation in field-based samples, particularly from rural areas [14]. This study tested the DESS and optimum sample to DESS ratios to preserve *S. ratti* DNA in canine feces for up to 56 days in the laboratory setting. 

The 1:3 sample to DESS ratio was further validated through the preservation of field-collected canine fecal samples stored at room temperature for a maximum of 30 days until being processed. The subsequent PCR results identified 17 canine fecal samples positive for *Strongyloides* spp. out of a total of 37 field-collected samples. The study demonstrated that DESS can preserve DNA in fecal samples, which presents a more challenging matrix containing complex polysaccharides, bile salts, urate, and lipids compared to soil or other tissues [9]. 

One of the limitation to this study was that it was not feasible to have comparable field-collected samples not treated with DESS for comparison of DNA degradation. However, the laboratory based study demonstrated the DESS DNA preservation effect compared to no-treatment, *S. ratti* spiked fecal samples. In the preliminary study, no feces were autoclaved to prevent any change in the amount of DNAses and RNAses present in the sample which would impact on the total amount of DNA degradation, thus making these samples comparable to the field-collected non-autoclaved fecal samples.

DESS presents a non-toxic preservative method for *Strongyloides* spp. DNA, and can be used for the detection of *Strongyloides* spp. DNA in fecal samples in both animal and human models. Studying the environmental phase of the parasite is important to be able to apply a combined approach of disease treatment, including environmental as well clinical control. The first step would be to look at the potential environmental sources of *Strongyloides* spp. such as canine feces, wastewater, or sewage. As such, it is important to be able to use DESS, a non-toxic, easy to apply method, to preserve the *Strongyloides* spp. DNA in the samples collected from remote or rural areas. 

The DESS method for the storage of fecal samples to prevent the degradation of DNA could also be applied to clinical samples to improve sensitivity. Given that microscopy tests are not always sensitive enough [20,21], and serology can result in false positives due to the risk of cross-reactivity with other antibodies [22,23], PCR detection should be conducted to avoid misdiagnosis. This is particularly important in settings where culture techniques are not possible or impractical. In fact, in a personal communication with a clinical diagnostic laboratory worker, there was a hyperinfection strongyloidiasis case where a human stool sample was collected and transported from a rural area, resulting in DNA degradation. The subsequent laboratory results for PCR returned high Ct values that would not have been treated as a positive result, were it not for the fact that the serology was also positive and there was significant eosinophilia, but negative microscopy, which can be due to low larval output in feces. This suggests that DNA degradation in human feces as a result of long postage times may result in potential false negative results and misdiagnosis [24].

## 5. Conclusions

*S. stercoralis* is a parasitic nematode of public health concern, which is able to reproduce in both the environment and in a host. Real-time PCR is the most feasible method for its detection in both clinical and environmental samples. However, the sensitivity of the PCR method can be affected by DNA degradation, which is particularly an issue when the majority of samples are coming from rural and remote locations. This study demonstrated that both 1:1 and 1:3 sample to DESS ratios can preserve DNA in fecal samples for up to 56 days at room temperature prior to the subsequent real-time PCR detection of *Strongyloides* spp. The method was validated using field-collected canine fecal samples, which were stored at room temperature for up to 30 days. The DESS preservation method requires no additional kits or certain storage temperatures and is cost-effective, which presents a feasible method for DNA preservation in field-collected animal feces, and potentially can be applied in detecting *S. stercoralis* in human feces for clinical diagnostics.
